# Characterization of complete lncRNAs transcriptome reveals the functional and clinical impact of lncRNAs in multiple myeloma

**DOI:** 10.1038/s41375-021-01147-y

**Published:** 2021-02-17

**Authors:** Arantxa Carrasco-Leon, Teresa Ezponda, Cem Meydan, Luis V. Valcárcel, Raquel Ordoñez, Marta Kulis, Leire Garate, Estíbaliz Miranda, Victor Segura, Elisabeth Guruceaga, Amaia Vilas-Zornoza, Diego Alignani, Marién Pascual, Ane Amundarain, Laura Castro-Labrador, Patxi San Martín-Uriz, Halima El-Omri, Ruba Y. Taha, Maria J. Calasanz, Francisco J. Planes, Bruno Paiva, Christopher E. Mason, Jesús F. San Miguel, José I. Martin-Subero, Ari Melnick, Felipe Prosper, Xabier Agirre

**Affiliations:** 1 Área de Oncología, Centro de Investigación Médica Aplicada (CIMA), Universidad de Navarra, IDISNA, Pamplona, Spain; 2 Centro de Investigación Biomédica en Red de Cáncer, CIBERONC, Pamplona, Spain; 3 Division of Hematology/Oncology, Department of Medicine, Weill Cornell Medical College, New York, NY USA; 4 Department of Physiology and Biophysics, Weill Cornell Medicine, New York, NY USA; 5 The Bin Talal Bin Abdulaziz Alsaud Institute for Computational Biomedicine, Weill Cornell Medicine, New York, NY USA; 6 University of Navarra, Tecnun School of Engineering, San Sebastián, Spain; 7 Fundació Clínic per la Recerca Biomèdica, Barcelona, Spain; 8 Institut d’Investigacions Biomèdiques August Pi I Sunyer (IDIBAPS), Barcelona, Spain; 9 Department of Bioinformatics, CIMA/UNAV/IDISNA, Pamplona, Spain; 10 Flow Cytometry Core, CIMA, Universidad de Navarra, Pamplona, Spain; 11 Department of Hematology & BMT, Hamad Medical Corporation, NCCCR, Doha, Qatar; 12 CIMA Lab Diagnostics, Universidad de Navarra, Pamplona, Spain; 13 Servicio de Hematología, Clínica Universidad de Navarra, Pamplona, Spain; 14 Institució Catalana de Recerca i Estudis Avançats (ICREA), Barcelona, Spain; 15 Departamento de Fundamentos Clínicos, Universitat de Barcelona, Barcelona, Spain

**Keywords:** Myeloma, Myeloma

## Abstract

Multiple myeloma (MM) is an incurable disease, whose clinical heterogeneity makes its management challenging, highlighting the need for biological features to guide improved therapies. Deregulation of specific long non-coding RNAs (lncRNAs) has been shown in MM, nevertheless, the complete lncRNA transcriptome has not yet been elucidated. In this work, we identified 40,511 novel lncRNAs in MM samples. lncRNAs accounted for 82% of the MM transcriptome and were more heterogeneously expressed than coding genes. A total of 10,351 overexpressed and 9,535 downregulated lncRNAs were identified in MM patients when compared with normal bone-marrow plasma cells. Transcriptional dynamics study of lncRNAs in the context of normal B-cell maturation revealed 989 lncRNAs with exclusive expression in MM, among which 89 showed de novo epigenomic activation. Knockdown studies on one of these lncRNAs, *SMILO* (specific myeloma intergenic long non-coding RNA), resulted in reduced proliferation and induction of apoptosis of MM cells, and activation of the interferon pathway. We also showed that the expression of lncRNAs, together with clinical and genetic risk alterations, stratified MM patients into several progression-free survival and overall survival groups. In summary, our global analysis of the lncRNAs transcriptome reveals the presence of specific lncRNAs associated with the biological and clinical behavior of the disease.

## Introduction

Multiple myeloma (MM) is a hematological neoplasm characterized by uncontrolled clonal proliferation of plasma cells (PCs) in the bone marrow. Despite advances in the therapy of this disease, which currently is associated with a median survival of 7 years, it is still considered an incurable malignancy, as most MM patients become resistant to treatment resulting in disease progression [1]. One of the main challenges of managing this disease is its clinical heterogeneity, featuring various subtypes and distinct outcomes. Studies of the molecular pathogenesis of MM have not completely elucidated the mechanisms underlying the aforesaid heterogeneity. Identification of such alterations would be critical in order to develop biomarkers to improve prognostic stratification of patients and to develop novel therapeutic targets for specific subgroups of patients. It has been suggested that genetic and/or epigenetic alterations underlie the MM clinical heterogeneity [2]. Such lesions not only affect the expression of coding genes, but also the expression of non-coding RNAs (ncRNAs), which are emerging as potential drivers and therapeutic targets of a variety of diseases [3].

The magnitude of the non-coding transcriptome in human cells is underlined by the fact that although around 90% of the genome is transcribed into RNA, only 1–2% is translated into proteins. It is now well accepted that ncRNAs play an essential role in cellular development, physiology, and pathology of human diseases [4]. Among these ncRNAs, long non-coding RNAs (lncRNAs) (>200 nt) are known to be involved in crucial functions such as gene expression modulation, chromatin reorganization, immune response, and cell differentiation [5–8], and their deregulation contributes to human carcinogenesis, metastasis, and even to chemotherapy resistance [9]. Thus, deregulation of the expression of lncRNAs can impact relevant pathways involved in the pathogenesis and/or progression of different types of cancers, including MM [3, 10–13].

In MM, altered expression of a small number of lncRNAs has been associated with the progression and survival of patients [14–17], suggesting that these elements play a role in the pathogenesis of the disease. Although high-throughput analyses characterizing the deregulation of annotated lncRNAs in MM have been published [18], comprehensive studies designed to investigate the complete lncRNAs transcriptome of the disease in the context of the maturation program of the B-cell lineage including both annotated and novel transcripts have not yet been performed. These types of analyses are now feasible due to the use of strand-specific whole transcriptome RNA-sequencing (ssRNA-seq), resulting in the identification and characterization of lncRNAs in multiple diseases, and therefore, could also be applied to MM [7, 19]. In the present work, we aimed at deciphering the entire lncRNAs transcriptome of MM using ssRNA-seq, hypothesizing that this approach will help us to better understand MM heterogeneity and would also provide novel clinical tools, including prognostic markers and therapeutic targets for the treatment of this disease.

## Methods

### Samples

Bone marrow aspiration specimens were obtained from 38 newly diagnosed untreated MM patients (Supplemental Table 1), and from three healthy donors. The data from normal B-cells (naive, memory, germinal center, centroblast, centrocytes, tonsil PCs (TPCs), and bone marrow PCs (BMPCs)) was generated by our laboratory as previously described [20]. All patients and healthy donors gave informed consent for their participation in this study, which was approved by the clinical research ethics committee of Clínica Universidad de Navarra. Details are described in Supplemental Methods.

### ssRNA-seq library preparation, sequencing, and analysis

Total RNA was isolated using TRIzol® Reagent (Life Technologies) and preparation, sequencing, analysis details, and annotation of lncRNAs from ssRNA-seq data are described in Supplemental Methods. ssRNA-seq data are available at GEO under accession number GSE151063. Transcripts expressed in MM are shown in Supplemental Table 2.

### Differential expression and heterogeneity analysis

To define differential expression between MM and BMPC samples a criterion of *B* > 3 was applied. Sample variability was studied using the coefficient of variation (CV). CVs were compared using a statistical test (*t*-test). Upregulated and downregulated lncRNAs in MM are described in Supplemental Table 3. The group of lncRNAs with a specific expression in MM is indicated in Supplemental Table 4. Details are described in Supplemental Methods.

### Chromatin histone marks analysis

Chromatin states of MM and B-cell populations were studied as described in Ordoñez et al. [21]. We defined 89 lncRNAs with de novo gain of chromatin marks in MM (Supplemental Table 5). Details are described in Supplemental Methods.

### Study and characterization of lncRNA *SMILO*

DNA methylation data of CpGs across *SMILO* (specific myeloma intergenic long non-coding RNA) promoter were obtained from previous data published by our group [22] (Supplemental Methods). *SMILO* knockdown was performed by the shRNA system. Knockdown effects were measured by analyzing MM cell proliferation by MTS assays and apoptosis by Annexin V-FITC assays. *SMILO* knockdown was also studied by Bulk RNA-seq. Libraries were sequenced in an Illumina NextSeq 500. MARS-seq data are available at GEO under accession number GSE134057. All processes are described in Supplemental Methods. All primer sequences for qPCR are described in Supplemental Table 6.

### Survival studies using the CoMMpass dataset

For progression-free survival (PFS) and overall survival (OS) analyses, we used the data from the IA14 release of the Multiple Myeloma Research Foundation (MMRF) CoMMpass study dataset. Details are described in Supplemental Methods.

## Results

### Characterization of the entire lncRNAs transcriptome of MM

**Fig. 1:**
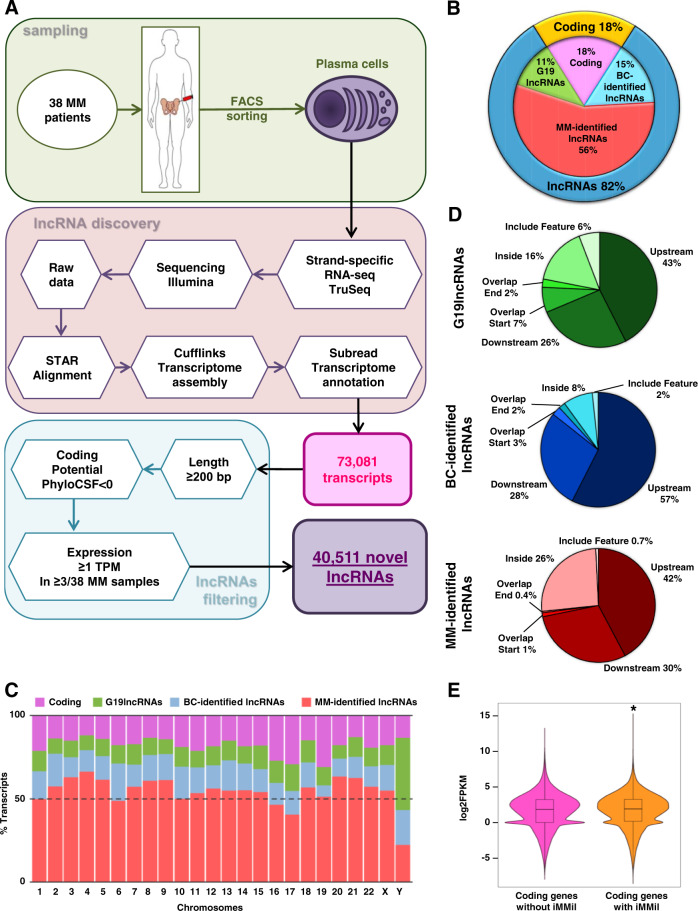
Complete characterization of the lncRNAs transcriptome of MM. **A** Schematic of the strategy used for ssRNA-seq data processing and for the identification of novel transcripts and lncRNAs in MM patients. **B** Pie chart representation of transcripts detected and expressed in at least 3 of the 38 MM patients with a minimum expression of 1 TPM. **C** Cumulative percentage of each type of expressed genes distributed by chromosome. **D** Pie charts representing the genetic location of G9lncRNAs, BC-identified lncRNAs, and MM-identified lncRNAs. **E** Graph showing the expression levels of coding genes harboring (right) or not (left) inside MM-identified lncRNAs (iMMil) (*p*-value = 6.857e^−14^). MM: multiple myeloma patients, G19lncRNAs: lncRNAs previously annotated in Gencode 19 database, BC-identified lncRNAs: lncRNAs identified in different B-cell subpopulations on our previous work, MM-identified lncRNAs: lncRNAs identified in MM patient samples, iMMil: inside MM-identified lncRNAs.

In order to fully characterize the transcriptome of MM, including all types of lncRNAs, we performed paired-end ssRNA-seq of PCs purified from the bone marrow of 38 MM patients (Supplemental Table 1). Transcriptome assembly of aligned reads demonstrated the presence of 73,081 novel transcripts in MM PCs. Such transcripts were filtered by length (>200 bp), low coding potential (PhyloCSF < 0), and expression level (≥1 TPM), leading to the identification of 40,511 novel lncRNAs that were expressed in at least 3 of the 38 MM patient samples (Fig. 1A; Supplemental Table 2). The expression of some of these novel lncRNAs was validated in new MM patient samples (Supplemental Fig. 1A). The comparison of the number of expressed coding and lncRNA genes in MM, with the latter including: (1) lncRNAs previously annotated in Gencode G19 (G19lncRNAs), (2) lncRNAs identified in different B-cell subpopulations in our previous work (BC-identified lncRNAs) [20], and (3) the set of novel lncRNAs identified in our MM patient samples (MM-identified lncRNAs), revealed that lncRNAs accounted for 82% of MM transcriptome, with coding transcripts representing only the 18% of the expressed transcripts in MM. The novel lncRNAs identified in MM comprised the largest group among the studied groups of lncRNAs (including those previously annotated), accounting for 56% of all expressed genes in MM PCs (Fig. 1B). In order to determine whether specific genomic areas of MM cells were associated with increased transcription of lncRNAs, we analyzed the genome-wide distribution of these elements, observing that coding and long non-coding genes were uniformly distributed among chromosomes (Fig. 1C; Supplemental Table 2). Next, lncRNAs were classified regarding to their distance to coding genes, showing that upstream transcripts were the most common type, followed by downstream lncRNAs, and lncRNAs located inside coding genes (Fig. 1D). Interestingly, lncRNAs identified in MM showed a higher percentage of lncRNAs located inside coding genes (26%) as compared to previously annotated lncRNAs (Fig. 1D). Furthermore, the expression of coding genes harboring such inside MM-identified lncRNAs (3,223 coding genes) was significantly higher than the rest of coding genes without inside MM-identified lncRNAs (9,724 coding genes) (*p*-value = 6.857e^−14^) (Fig. 1E), suggesting that the increased expression of specific coding genes could trigger the regulation of a subset of lncRNAs in MM cells or vice versa. These results suggest that both coding and lncRNA genes, possibly together and encoded from the same regions of the genome, may be key participants of tumor development. Accordingly, among such genes with inside lncRNAs, we observed relevant genes with a known role in MM pathogenesis, such as *IRF4, FGFR3*, and *SLAMF7*. Overall, these results indicate that the MM transcriptome is more complex and extensive than previously appreciated and that lncRNAs represent its vast majority.

### Heterogeneity and specificity of lncRNAs expression in MM

**Fig. 2:**
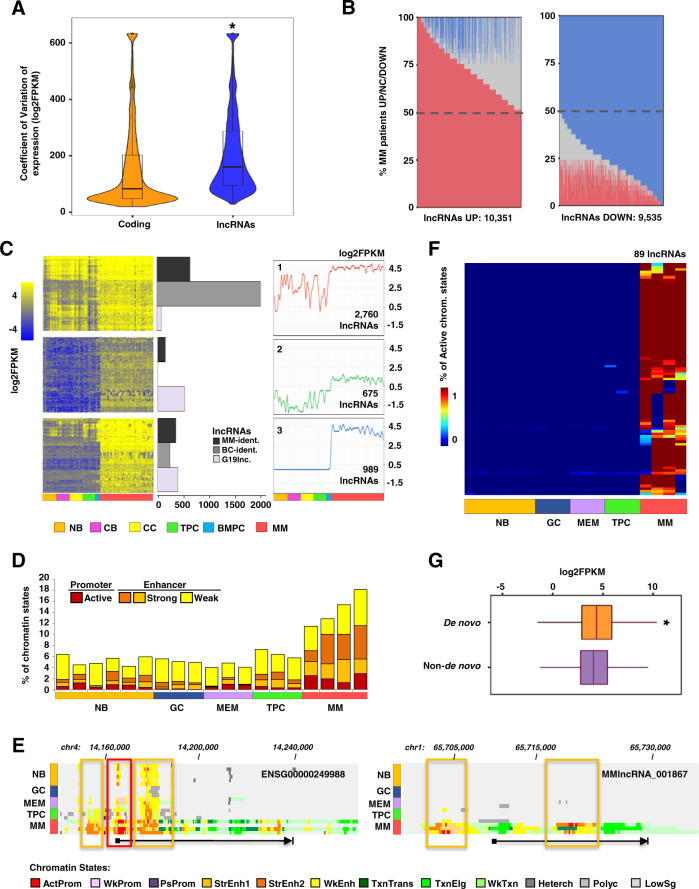
lncRNAs show a heterogeneous and dynamic expression among MM patients. **A** Violin plots representing the coefficient of variation of the expression of coding and lncRNA transcripts in all MM samples (*p*-value < 2.2e^−16^). **B** Analysis of expression heterogeneity of lncRNAs in MM patient samples. Barplot of the percentage of MM patients (*y* axis) that show overexpression (red), downregulation (blue), or no significant changes (gray) for each lncRNAs (*x* axis). **C** Expression of lncRNAs from the three transcriptional dynamisms detected along with B-cell differentiation and in MM patient samples. For each dynamism, a heatmap showing the RNA-seq expression of the lncRNAs (left), the number of each type of lncRNA (center), and the centroid (expression average) (right) in normal B-cell subpopulations and MM patient samples are depicted. **D** Chart depicting the percentage of the length of lncRNAs (*y* axis) occupied by promoter and enhancer chromatin marks of the 989 lncRNAs from cluster three in normal B-cell subpopulations and MM patient samples. **E** Genome browser snapshots showing chromatin states of two loci of MM-specific lncRNAs. Red and orange boxes encompass the gain of the active promoter and strong enhancer chromatin marks, respectively, in MM compared to B-cell populations. Each chromatin state is represented by one color. The arrow indicates the length and direction of expression of the lncRNAs. **F** Heatmap showing de novo activation of lncRNAs in MM. The color scale indicates the percentage of active chromatin sates in the promoter region of each lncRNA. **G** Box plot representing the expression level of lncRNAs showing de novo active epigenetic marks in MM patients (orange) and those without de novo gain (purple) (*p*-value = 3.724e^−07^). CV: coefficient of variation of the expression, MM: multiple myeloma patients, MM-ident.: lncRNAs identified in MM patient samples, BC-ident.: lncRNAs identified in different B-cell subpopulations on our previous work, G19lnc.: lncRNAs previously annotated in Gencode 19 database, NB: naïve, GC: germinal center, CB: centroblast, CC: centrocyte, MEM: memory B-cell, TPC: tonsil plasma cell, BMPC: bone marrow plasma cell, Chr: chromosome, ActProm: active promoter, WkProm: weak promoter, PsProm: poised promoter, StrEnh1: strong enhancer 1, StrEnh2: strong enhancer 2, WkEnh: weak enhancer, TxnTrans: transcription transition, TxnElg: transcription elongation, WkTxn: weak transcription, Heterch: heterochromatin, Polyc: polycomb, LowSg: low signal, De novo: lncRNAs with de novo chromatin active marks, Non-de novo: lncRNAs without de novo chromatin active marks.

Next, we compared the lncRNAs transcriptome between MM and normal PCs isolated from the bone marrow (BMPC) of healthy donors. Differential gene expression analysis comparing MM and BMPC samples demonstrated that despite the large number of lncRNAs identified in MM specimens, only 571 lncRNAs and 78 coding genes were differentially expressed (*B* > 3). To determine whether the relatively small number of differential transcripts could be due to highly heterogeneous gene expression levels of MM PCs, we analyzed the CV of lncRNAs and coding genes in MM PCs and BMPCs. We detected a greater degree of expression heterogeneity in MM than in BMPCs for all types of transcripts (Supplemental Fig. 1B). Interestingly, the heterogeneity of expression in MM samples was significantly higher for lncRNAs than for coding genes (Fig. 2A; Supplemental Fig. 1B), a finding that may explain the low number of differentially expressed lncRNAs detected, and which suggests that these elements may contribute to the clinical heterogeneity of the disease. In order to detect aberrantly expressed lncRNAs in a manner that would account for such heterogeneity, we individually compared the expression profile of each MM patient to the profile of BMPCs. We observed that some lncRNAs were overexpressed or downregulated in a very high percentage of patients (>80%), while others were altered in a small number of samples. For further analyses, we selected those lncRNAs that were overexpressed or downregulated in at least 50% of the patients, and that showed the opposite direction of deregulation in less than 25% of the individuals. Using these criteria, we identified 10,351 overexpressed and 9,535 downregulated lncRNAs in MM patients (Fig. 2B; Supplemental Table 3). Among them, we detected lncRNAs as *MALAT1*, described in previous MM studies [11]. We also validated some of the differentially expressed lncRNAs in a new series of MM patients (Supplemental Fig. 1C).

Next, we aimed to identify, from the previous analysis, the subset of lncRNAs that were dynamically deregulated in MM PCs in the context of B-cell differentiation, as they could potentially represent specific therapeutic targets for the disease. For this purpose, the expression of these 19,886 lncRNAs was analyzed in different normal subpopulations of B-cell differentiation states including naïve, centroblasts, centrocytes, TPCs, and BMPCs, and compared with the expression in MM PCs. Three different patterns of expression of lncRNAs were observed (Fig. 2C). Cluster 1 comprised 2,760 lncRNAs with an irregular expression pattern along with B-cell differentiation and a uniformly high expression in MM PCs. Cluster 2 contained 675 lncRNAs with low expression throughout B-cell differentiation and a slight increase in MM PCs. Finally, cluster 3 (Supplemental Table 4) showed a very low and homogeneous expression of 989 lncRNAs throughout B-cell differentiation with a clear increase in MM samples. This last pattern of expression suggests the existence of a group of lncRNAs almost exclusively expressed in MM PCs (named as MM-specific lncRNAs). Based on their specific expression in MM PC, we focused on this subgroup of lncRNAs for additional analyses.

### Regulation of MM-specific lncRNAs

In order to determine whether the expression of MM-specific lncRNAs in MM was epigenetically regulated, ChIP-seq data of six histone marks from our previous work [21], defining common chromatin states, were analyzed (H3K4me3, H3K4me1, H3K27ac, H3K36me3, H3K27me3, and H3K9me3) [23]. As the gain of active epigenetic marks has been used to discriminate lncRNAs from transcriptional noise [23], this analysis helped us to further corroborate our findings.

A global increase of active histone marks at the loci of MM-specific lncRNAs in MM when compared to normal B-cell subpopulations was observed (Fig. 2D; Supplemental Fig. 2A), and was mainly related to both active promoters and enhancers (Fig. 2E; Supplemental Fig. 2A). This is in agreement with several studies showing that lncRNAs can be transcribed from promoter or enhancer regions of the genome [24]. Although the majority of MM-specific lncRNAs showed an increase of active chromatin marks, only a small subset of these lncRNAs (89 of 989) presented a de novo chromatin activation, in which repressive marks present in subpopulations of normal B-cells were replaced by activating chromatin modifications in MM specimens (Fig. 2F; Supplemental Fig. 2B; Supplemental Table 5). Interestingly, the expression of these 89 lncRNAs showing de novo epigenetic activation (Fig. 2F) was significantly higher than the other MM-specific lncRNAs (Fig. 2G; Supplemental Fig. 2B, C). Altogether, these data suggest an epigenetic rewiring in MM through two different ways: (1) the loci of most MM-specific lncRNAs are in a partially active or poised chromatin state in normal B-cells and become completely active in MM and (2) the loci of a small subset of MM-specific lncRNAs are inactive in normal cells and undergo a de novo epigenetic activation in the disease, leading to an aberrant upregulation of these elements.

### MM-specific lncRNA *SMILO* is essential for the survival of MM cells

**Fig. 3:**
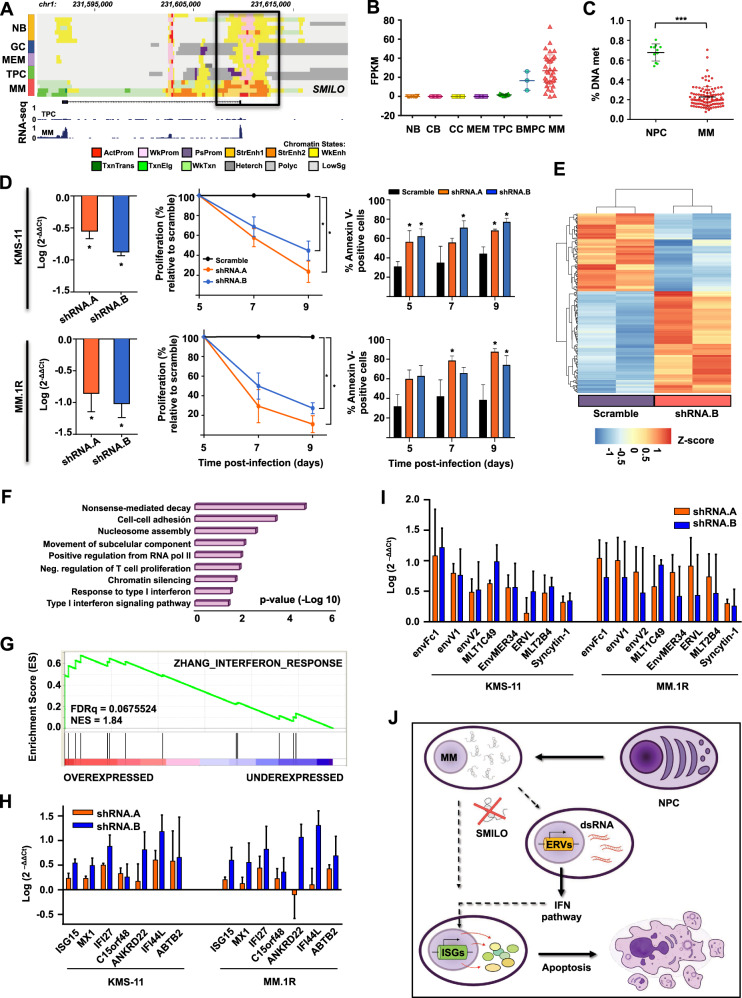
*SMILO* is essential for the survival of MM cells. **A** Genome browser snapshot showing chromatin states representation and RNA-seq levels of *SMILO* locus in normal B-cell populations and MM patient samples. The black box indicates the promoter region of *SMILO*, showing the gain of chromatin active marks such as promoter and strong enhancer marks in MMs. Each chromatin state is represented by one color. **B** *SMILO* expression obtained from strand specific RNA-seq data performed in several subpopulations of B-cell differentiation and MM patient samples. FPKM values are shown for each sample. **C** Percentage of DNA methylation of a CpG (cg08458637) located in the promoter region of *SMILO* obtained from a DNA methylation array data performed in NPCs and MM patients in our previous study [35]. **D** Knockdown of *SMILO* by two different shRNAs in KMS-11 and MM.1R MM cell lines. Levels of *SMILO* expression were determined by qPCR (left). Gene expression normalized to *GUSß* is presented relative to that observed in cells infected with a scrambled shRNA. Proliferation curves (center) and the percentage of annexin-V positive cells (right) were detected at the indicated times after infection. Scramble represented in black, shRNA.A in orange and shRNA.B in blue. The average of three independent biological replicates ±SD is shown. **E** Heatmap showing the RNA-seq data of 194 differentially expressed genes upon *SMILO* knockdown in KMS-11 cell line. **F** Gene ontology (GO) analysis showing the top GO terms of the differentially expressed genes after *SMILO* knockdown in KMS-11 cells. **G** GSEA plot of the IFN pathway identified comparing KMS-11 cells with or without *SMILO* knockdown. **H**–**I** Validation by qPCR of the overexpression of *ISGs* (**H**) or *ERVs* (**I**) after inhibition of *SMILO* in KMS-11 and MM.1R cells. Samples were collected after 5 days of infection. Gene expression normalized to *GUSß* is presented relative to that observed in cells infected with a scrambled shRNA. The average of three independent biological replicates ±SD is shown. **J** Schematic representation of the putative role of *SMILO* knockdown in the promotion of MM cell death. Chr: chromosome, NB: naïve, GC: germinal center, MEM: memory B-cell, TPC: tonsil plasma cell, MM: multiple myeloma patient, ActProm: active promoter, WkProm: weak promoter, PsProm: poised promoter, StrEnh1: strong enhancer 1, StrEnh2: strong enhancer 2, WkEnh: weak enhancer, TxnTrans: transcription transition, TxnElg: transcription elongation, WkTxn: weak transcription, Heterch: heterochromatin, Polyc: polycomb, LowSg: low signal, CB: centroblast, CC: centrocyte, BMPC: bone marrow plasma cell, NPC: normal plasma cell, % DNA met: percentage of DNA methylation, FDRq: false discovery rate, NES: normalized enrichment score, ERVs: endogenous retroviruses genes, ISGs: interferon-stimulated genes, IFN pathway: interferon pathway, dsRNA: double-strand RNA.

Among the 89 lncRNAs expressed from de novo epigenomically activated regions in MM, we identified *LINC00582* (*ENSG00000229228*, named *SMILO*) (Fig. 3A), and intergenic lncRNA composed of two exons, located between *TSNAX* and *DISC1* coding genes, and transcribed from the negative strand of chromosome band 1q42.2, a genomic region frequently amplified in MM patients. *SMILO* expression was not detectable throughout B-cell differentiation, except for marginal expression levels in some BMPCs (Fig. 3B) and was upregulated in 64% of MM patients when compared to BMPCs. Expression of *SMILO* was significantly higher in patients with 1q amplification, although this increased expression was not exclusive of this group of patients (Supplemental Fig. 3), suggesting that other causes besides this genetic lesion may trigger its deregulation. The de novo epigenomic activation of the *SMILO* locus was associated with a loss of DNA methylation in MM PCs in contrast with normal PCs (Fig. 3C; Supplemental Fig. 4A). These results strongly suggested that, besides 1q amplification, epigenetic mechanisms are involved in the activation of *SMILO* and its overexpression in MM patients.

Knockdown of *SMILO* using two different shRNAs resulted in a decrease in the proliferation rate of three MM cell lines and an increase in the percentage of apoptotic cells (Fig. 3D; Supplemental Fig. 4B), indicating that *SMILO overexpression * is essential for the survival of MM cells. RNA-seq analysis of our *SMILO* knockdown system in KMS-11 cells showed a downregulation and upregulation of 84 and 110 genes, respectively (Fig. 3E). Coding genes deregulated upon *SMILO* knockdown were enriched in several processes regulating gene expressions such as nucleosome assembly, nonsense-mediated decay or chromatin silencing, and in relevant known functions and pathways for MM cells, such as cell adhesion (Fig. 3F). Interestingly, one of the top enriched pathways for the up-regulated genes after inhibition of *SMILO* expression was the type I Interferon (IFN) signaling pathway (Fig. 3G; Supplemental Fig. 4C), whose deregulation has proven to be key for the homeostasis of MM cells. In addition, knockdown of *SMILO* led to upregulation of several interferon-stimulated genes (ISGs—*ISG15, IFI27*, and *MX1*), suggesting that *SMILO* upregulation in MM maintains these coding genes repressed, resulting in anti-apoptotic and proliferative effects for the MM cell. These results were further validated by qPCR in two additional myeloma cell lines (Fig. 3H; Supplemental Fig. 4D). The involvement of the IFN pathway in the cell death of MM cells was proven by adding different concentrations of IFN alpha (IFNα) to MM.1S, MM.1R, and KMS-11 MM cell lines (Supplemental Methods). The use of IFNα triggered an increase in apoptosis, a decrease in cell proliferation and upregulation of different ISGs (Supplemental Fig. 4E–G). Furthermore, expression of endogenous retroviruses (ERVs), known activators of ISGs [25], was upregulated upon inhibition of *SMILO* (Fig. 3I; Supplemental Fig. 4H), suggesting that these elements could be responsible for the activation of the IFN pathway. Altogether, our data indicate that *SMILO* overexpression is necessary for the survival of MM cells and its inhibition could trigger the overexpression of ERVs and the activation of the IFN pathway ultimately leading to the induction of cell-autonomous death potentially through immunogenic cell death (Fig. 3J).

### Prognostic value of MM-specific lncRNAs

**Fig. 4:**
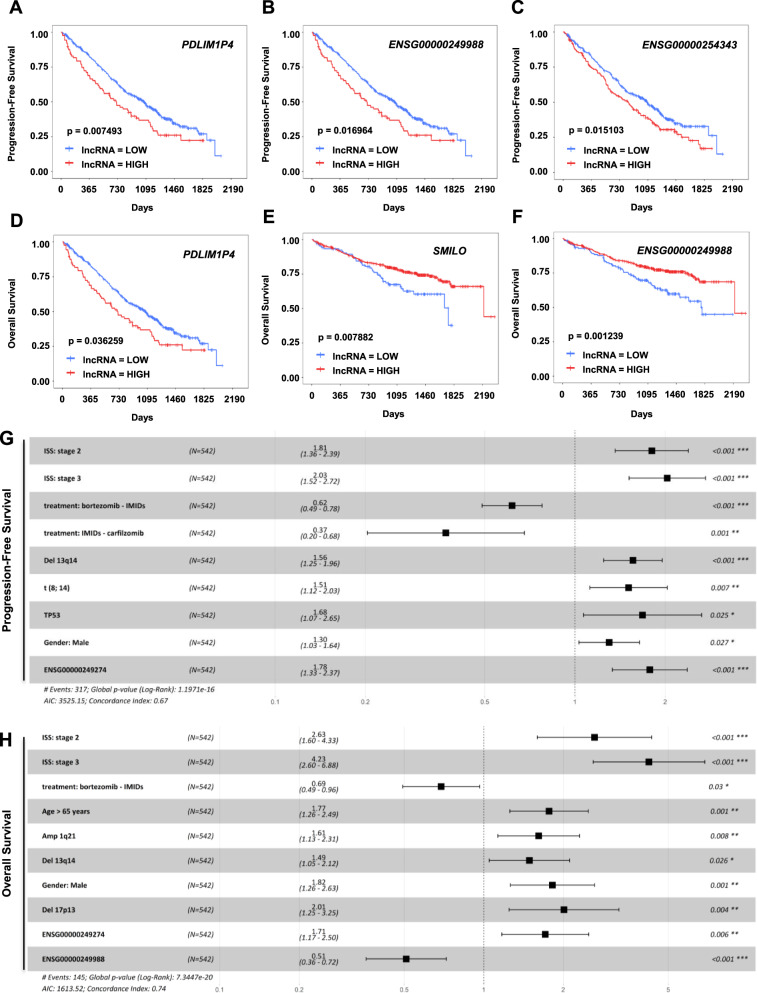
Prognostic value of lncRNAs in MM. **A**–**C** Survival analysis of *PDLIM1P4*, *ENSG00000249988*, and *ENSG00000254343* performed with CoMMpass dataset showing PFS of MM patients. Kaplan–Meier curves represent a bi-level state expression (high and low) of the lncRNA. **D**–**F** Survival analysis of *PDLIM1P4*, *SMILO*, and *ENSG00000249988* performed with CoMMpass dataset showing OS of MM patients. **G**–**H** Multivariate analyses evaluating the significance of the different genetic and clinical factors together with the expression of *PDLIM1P4* in PFS (**G**), and *PDLIM1P4*, and *ENSG00000249988* in OS (**H**), respectively. LOW: low expression of the lncRNA, HIGH: high expression of the lncRNA.

Based on the highly heterogeneous expression of lncRNAs among MM patients, we finally aimed to determine whether the expression of MM-specific lncRNAs might have prognostic value in MM patients. For this purpose, we used RNA-seq data from 542 patients included in the IA14 CoMMpass study and from whom clinical information was available to analyze PFS and OS. As RNA-seq data included in CoMMpass could only provide reliable information regarding previously annotated lncRNAs, we restricted our analysis to 7 out of the 89 de novo-activated MM-specific lncRNAs annotated in Gencode. Expression levels of 6 out these 7 lncRNAs were detected in samples included in the CoMMpass study: *ANKRD20A5P* (*ENSG00000186481*), *SMILO*, *PDLIM1P4* (*ENSG00000249274*), *ENSG00000249988*, *ENSG00000254343,* and *RHOT1P1* (*ENSG00000266145*) (Supplemental Fig. 5A). Interestingly, expression of *ANKRD20A5P, SMILO, ENSG00000254343*, and *RHOT1P1* were significantly associated with the presence of amp(1q), while expression of *PDLIM1P4* and *ENSG00000249988* did not show any significant association with different MM genetic groups (Supplemental Fig. 3). To assess whether the expression of MM-specific lncRNAs could be associated with the prognosis of MM patients, we analyzed the PFS and OS of these patients according to the level of expression of each lncRNA, dicotomizing cases into two groups based on the expression levels (high or low; Supplemental Fig. 5B). Firstly, we performed a univariate statistical survival analysis. We observed that the expression of *PDLIM1P4*, *ENSG00000249988*, and *ENSG00000254343* (*p*-value = 0.007493, 0.016964, and 0.015103, respectively) was associated with PFS, dividing MM patients into two risk factor groups (Fig. 4A–C; Supplemental Fig. 6A–C). In the case of OS analysis, the expression of *PDLIM1P4, SMILO*, and *ENSG00000249988* (*p*-value = 0.036259, 0.007882, and 0.001239, respectively) showed statistically significant results (Fig. 4D–F; Supplemental Fig. 6D–F). After the univariate analysis, we performed a multivariate statistical analysis with those lncRNAs with significantly results from the univariate analysis, and the different clinical and genetic alterations that also appeared with statistically significant results for PFS and OS: ISS, treatments, del(13q), amp(1q), t(8, 14), TP53, age over 65 years and gender for PFS analysis; and ISS, treatments (Bortezomib-IMIDs and Carfilzomib-IMIDs), age over 65 years, high-risk factors, amp(1q), del(17p), del(1p), del(13q), t(14, 20), TP53, gender and race for OS analysis. In this case, we detected that the high expression of *PDLIM1P4* and the factors Stage 2 and 3 of ISS, del(13q), t(8, 14), TP53, gender male, and treatments with Bortezomib-IMIDs and Carfilzomib-IMIDs resulted in statistically significant for PFS (Fig. 4G). We observed that in PFS analyses, the use of Bortezomib with IMIDs and Carfilzomib with IMIDs confer a good prognosis for MM patients. For the OS multivariate analysis we used the combination of the different risk factors together with the expression of the three lncRNAs that showed significant results in the univariate analysis. This analysis revealed that high expression of *PDLIM1P4* and *ENSG00000249988* together with Stage 2 and 3 of ISS, treatment with Bortezomib-IMIDs, age over 65 years, amp(1q), del(13q), del(17p) and gender male could stratify MM patients into different risk groups (Fig. 4H). Overexpression of *ENSG00000249988* and the use of Bortezomib with IMIDs was associated with  longer OS. Finally, we also performed an ANOVA test to compare the models derived from clinical and genetic high-risk factors alone, or in combination with the expression of lncRNAs, finding a significant improvement for the second condition in both PFS (*p*-value = 0.0002) and OS (*p*-value = 0.0001).

These results demonstrate that a combination of the expression of MM-specific lncRNAs with established genetic biomarkers could have an important impact on the prognosis of patients with MM.

## Discussion

Our study proposes a systematic approach to globally characterize the role of lncRNAs in MM and identify epigenetically-regulated transcripts with functional and clinical value. We discovered 40,511 novel lncRNAs expressed in PCs of MM patients, notably increasing the number of previously annotated and expressed lncRNAs in this disease [18, 26]. Furthermore, we demonstrate that 82% of the distinct transcripts detected in MM were lncRNAs, suggesting that these lncRNAs could play an important role in this disease. These elements were more diverse and heterogeneously expressed than coding genes and some of them were transcribed from the internal regions of relevant coding genes implicated in the pathogenesis of MM. These results place lncRNAs as another factor to be taken into account, together with genetic and epigenetic alterations, in the study of pathogenesis and clinical behavior of MM [21, 27–29]. So far, few studies have decoded the complete lncRNAs transcriptome of different cancers [6, 30, 31]. Similar to our work, these studies demonstrate that lncRNAs comprise an important fraction of the expressed transcriptome, and as such, represent an underexploited source of novel cancer-related biomarkers.

By including RNA-seq data of different normal B-cell subpopulations (from naïve B-cell to BMPC) we could identify 989 lncRNAs showing a specific expression in PCs of MM patient samples. These results indicate that lncRNA expression not only shows cell-type specificity [20, 32] but also can be tumor-specific. In addition, the majority of MM-specific lncRNAs were associated in MM PCs with a gain of active histone marks defining active promoters or enhancers [21, 33]. On the one hand, the gain of promoter and enhancer marks at the loci of MM-specific lncRNAs validates them as real lncRNAs and not transcriptional noise, showing that this group of lncRNAs was composed of both enhancer and promoter-derived lncRNAs [20, 34]. While most lncRNAs from this group presented a poised or partially active chromatin state in normal B-cell populations that became active in MM, a small group of 89 lncRNAs presented de novo epigenetic activation in MM. In this group, repressing histone modifications during B-cell differentiation were replaced by active marks in MM. Intriguingly, lncRNAs showing de novo epigenetic activation presented significantly higher expression levels than other MM-specific lncRNAs. These results suggest that such loci may need tighter epigenetic regulation in order to ensure their physiological repression in normal cells. Further studies will be needed in order to ascertain the mechanisms that trigger the epigenetic deregulation of MM-specific lncRNAs, but probably different mechanisms play a role. These include activation of chromatin remodelers to make regions more accessible, chromatin activator complexes that remove repressive chromatin marks and place activating modifications at promoters and enhancers, as well as transcription factors, that collaborate in order to promote an epigenetic rewiring that ultimately leads to the overexpression of these group of lncRNAs in MM [35, 36].

We showed that one of those de novo epigenetically activated MM-specific lncRNA, *SMILO*, is essential for the survival of MM cells. We showed that inhibition of *SMILO* expression in MM cells triggered the overexpression of ERVs and ISGs, leading to the activation of the IFN pathway and, as a consequence, to cell death. These results suggest that inhibition of *SMILO* potentially leads to the induction of cell-autonomous and could also produce immunogenic cell death in MM cells. Interestingly, similar overexpression of ERVs and ISGs and promotion towards an immunogenic cell death has been detected after the treatment of different types of tumor cells with epigenetic drugs, including inhibitors of DNA methyltransferases (DNMTs) [37], or protein lysine methyltransferases such as G9a [38]. These results suggest that *SMILO* could represent a relevant therapeutic target for the treatment of patients with MM. Moreover, its inhibition could have a synergistic effect with epigenetic drugs in preparating the tumor cell for immunogenic cell death. This could represent an attractive therapeutic strategy for its subsequent combination with immunotherapy, which is providing very encouraging results in the treatment of MM [39, 40].

Finally, we also identified MM-specific lncRNAs as prognostic biomarkers that improve the stratification of MM patients. Up to date, genetic alterations are the only well established genomic parameters used to stratify the clinical outcome of MM [27, 29, 41]. However, increasing amount of evidence suggests that lncRNAs could also be used as risk factors to assess the clinical course of MM [18]. In the multivariate statistical analysis, we showed that the expression of *PDLIM1P4* together with 8 clinical and genetic risk factors divided MM patients into different level risk groups for PFS. In the case of OS, two lncRNAs, *PDLMI1P4* and *ENSG00000249988*, together with 8 different clinical and genetic risk factors stratified MM patients into different OS risk groups. These results indicate that incorporation of the expression of lncRNAs to the standards of traditional clnical and genetic risk factors could improve the identification of MM patients with different prognosis. Future studies will determine whether MM-specific lncRNAs can also help stratify patients in groups showing different treatment responses.

Taken together, our study provides a comprehensive picture of the lncRNAs transcriptome of MM, showing that these non-coding elements are heterogeneously, dynamically, specifically expressed, and, in some cases, de novo activated in MM cells. Furthermore, we show that lncRNAs may play an important role in the pathogenesis of MM, and more importantly, they could be of relevant clinical use as prognostic biomarkers or even as therapeutic targets that could ultimately improve the outcome of MM patients.

## Supplementary information

Supplemental data

Supplemental Figures

Supplemental Table 1

Supplemental Table 2

Supplemental Table 3

Supplemental Table 4

Supplemental Table 5

Supplemental Table 6
