# Supplementary material for: Characterization of complete lncRNAs transcriptome reveals the functional and clinical impact of lncRNAs in multiple myeloma
Source: Leukemia. 2021 Feb 17;35(5):1438–50. doi: 10.1038/s41375-021-01147-y (PMC8102198; doi:10.1038/s41375-021-01147-y)
Supplement: Supplementary file 1 — Supplemental data [file 41375_2021_1147_MOESM1_ESM.docx]

**SUPPLEMENTAL METHODS**

***Samples***

Bone marrow aspiration specimens were obtained from 38 newly diagnosed untreated MM patients (**Supplemental Table 1**), and from 3 healthy donors. Plasma cells were purified by CD138+ positive magnetic separation using AutoMACS system (AutoMACS Pro Separator, Miltenyi Biotech), obtaining over 90% purity in all cases, as assessed by flow cytometry.

***ssRNA-seq library preparation and sequencing***

Total RNA was isolated using TRIzol® Reagent extraction method (Life Technologies), purified by RNeasy MinElute spin column (Qiagen) and treated with DNase I (Thermo Fisher) following the manufacturer's instructions. Total RNA was quantified using NanoDrop ND-1000 Spectrophotometer (Thermo Fisher). High quality purified total RNA (300-500 ng) from each sample was used for library preparation according to the user´s manual of Truseq Stranded Total ribo-depleted RNA sample preparation kit (Illumina). Library quality was assessed using Agilent 2100 Bioanalyzer (Agilent Technologies) and quantity was determined using Qubit (Life Technologies). Strand specific RNA libraries were multiplexed (6 samples per lane) and the sequencing was done with Illumina HiSeq 2500 (Illumina) as 50 base paired-end runs.

***ssRNA-seq data analysis***

A two-step alignment procedure was performed with STAR v2.4^1^ to remove any potential ribosomal RNA leftover from the ribodepletion step. A first pass alignment was done against human ribosomal sequences allowing multi-mapping. Unmapped reads from the first step were then aligned to hg19 human reference using GENCODE v19^2^ junction points. Cufflinks v2.2.1^3,4^ was run on the resulting alignment files to create *de novo* transcriptome assembly specific to each sample using strand-specific settings and GENCODE v19 as a database. Cufflinks outputs for all the samples were then merged with themselves and GENCODE database using cuff merge. The resulting assembly was then filtered to only novel intergenic and antisense transcripts, removing isoforms for known genes as well as novel intronic sense-overlapping transcripts due to challenges in separating them from transcription artifacts. R^5^ and GNU parallel^6^ was used to facilitate the analysis. The novel transcripts were then filtered for minimum length of 200. Coding potential of each transcript was checked using phyloCSF^7^ on all 3 ORFs of the transcript strand. Maximum score along the length of the transcript across all 3 ORFs was used as the maximum coding potential, novel transcripts that had a coding potential score greater than 0 were filtered out. Subread feature Counts^8^ was used to annotate each sample to the merged transcript annotation. Resulting counts were normalized to library-size using number of mapped reads to each sample by DESeq2,^9^ and converted into Transcripts-per-million (TPM).^10^ Any transcript (including coding genes, lncRNAs and novel transcripts) that was expressed in at least 3 samples with ≥1 TPM was included for the study (**Supplemental Table 2**). ssRNA-seq data are available at GEO under accession number GSE151063.

***Annotation of lncRNAs***

Before any analysis we annotated the position of the lncRNAs in the genome (both known and novel lncRNAs discovered in B-cells and MM patients) using the protein coding genes of Gencode version 19 as reference and the R/Bioconductor package ChIPpeakAnno.^11^ We considered the following categories: “Upstream”, “Downstream”, “Overlap”, “Overlap Start”, “Overlap End”, “Inside” and “Include Feature”. Based on this result, we compared the expression values of those protein-coding genes which have a MM-identified lncRNA “inside” with those protein coding genes without any MM-identified lncRNA “inside” using a statistical test (t-test).

***Differential expression analysis***

The analysis of differential expression was performed using the log2FPKM values. Quality control of the samples was performed using the sample hierarchical clustering, the correlation matrix of the samples and the PCA reduction of dimensionality method. One outlier sample was detected, so we removed this sample from the study. Then, a filtering process to eliminate low expressed genes was carried out, from which 18,578 protein coding genes and 71,797 lncRNAs were obtained for further analysis. The detection of differentially expressed genes was carried out using the R/Bioconductor limma package^12^ with the protein coding genes, the novel lncRNAs (BC-identified and MM-identified lncRNAs) and the known lncRNAs (G19lncRNAs) with more than 200 bps and annotated with biotypes “3 prime overlapping ncRNA”, “antisense”, “lincRNA”, “sense intronic”, “sense overlapping”, “pseudogene” or “processed transcript” in Gencode v19. For the contrast of interest (the comparison between MM samples and BMPC samples) we applied a criterion of B>3 to define differential expression.

***Sample heterogeneity study and fold-change based selection of gene expression changes***

The study of the sample variability was conducted using the coefficient of variation (CV). We compared the CV using a statistical test (t-test) of protein coding genes and all lncRNAs in all the samples. We followed another approach to identify those lncRNAs whose expression change in a certain percentage of the samples based on their levels of logFC with respect to the mean expression value of the available BMPC samples. We classified the lncRNAs as up-regulated (those with a logFC greater than 1 in at least 50% of the samples and lower than -1 in less than 25% of the samples), down-regulated (those with a logFC lesser than -1 in at least 50% of the samples and greater than 1 in less than 25% of the samples), and no-change (the rest of the lncRNAs). Upregulated and downregulated lncRNAs in MM are described in **Supplemental Table 3**.

The expression profiles from B-cell differentiation to MM of the genes selected using the previous method (up-regulated and downregulated) was further analyzed using a SOM neural network^13^ implemented using the Kohonen package of R with dimension 4x4 (16 clusters) and 2000 iterations. We selected the dimension of the network empirically using the number of clusters observed in the sample hierarchical clustering and the results of some iteration of the algorithm using different sizes in order to avoid clusters without elements or with more than one representative gene expression profile. We confirmed the convergence of the algorithm following the error rate and the clusters with similar centroid profiles were joined based on the hierarchical clustering of the centroids fixing the new number of clusters (n=10). Finally, a manual curation of the remaining clusters was performed to select the more representative clusters using a new hierarchical clustering of the centroids. We found 4 clusters, one with low variability expression profiles that was removed and 3 clusters with changes in expression along the experimental conditions. The group of lncRNAs with a specific expression in MM (cluster 3) is indicated in **Supplemental Table 4**.

***Chromatin histone marks analysis***

To investigate the chromatin landscape of lncRNAs of interest, we employed the epigenetic data and the genome segmentation into chromatin states of MM and B-cell populations as described in Ordoñez et al^14^. We excluded lncRNAs located in X and Y chromosomes and analyzed the percentage of active chromatin states (i.e. active promoter, strong enhancers, transcription transition, elongation and weak transcription) as compared to percentage of repressed chromatin states (i.e. weak and poised promoters, weak enhancers and heterochromatin) within the body of lncRNAs of interest (n=989), both in MM and normal B-cells. With that, we performed t-test, to select lncRNAs with differential percentage of active chromatin states in MM vs normal B-cells (FDR < 0.2). Afterwards, in order to obtain more refined list of lncRNAs, with homogenous and clearly different chromatin activity landscape in MM and normal B-cells, we included two additional more stringent filters: we set the absolute difference of mean chromatin activity between MM and all B-cells, and in particular between MM and TPCs, to be higher than 0.5. From this analysis we obtained 131 lncRNAs, all of them representing more active chromatin landscape in MM as compared to normal B-cells. Finally, from this subset of lncRNAs, we defined 89 that seem to be repressed in normal counterparts (maximum percentage of active chromatin states not higher than 25%) and gain *de novo* chromatin activation in MM (**Supplemental Table 5**).

***Study of lncRNAs expression and epigenetic activation marks***

This analysis was completed using a t-test to evaluate the difference in expression values between the lncRNAs of the cluster of interest with a gain in *de novo* chromatin activation marks and the lncRNAs of the cluster of interest without a gain in *de novo* chromatin activation marks.

***DNA methylation***

Methylation data of CpGs across *SMILO* promoter were obtained from previous data published by our group^15^. DNA methylation levels were validated using bisulfite pyrosequencing analysis. Genomic DNA was extracted using the NucleoSpin Tissue kit (Macherey Nagel) following the manufacturer’s instructions. One microgram of DNA was bisulfite-modified using the CpGenome DNA modification Kit (Chemicon International). For PCR amplification of the regions of interest, a “hot start” PCR (PyroMark PCR Kit, Qiagen) was used with the following protocol: 95 °C for 15 minutes, 45 cycles of 94 °C for 1 minute, 53 °C for 1 minute, and 72 °C for 1 minute, followed by a final 10 min extension at 72 °C. PCR was performed using 2 µl of modified DNA and a final concentration of 0.2 μM of each primer (**Supplemental Table 6**). Biotinylated PCR products were bound to Streptavidin Sepharose High Performance Beads (GE Healthcare) and processed to yield high quality ssDNA using the PyroMark Vacuum Prep Workstation (Biotage). Pyrosequencing reactions were performed using the PyromarkTM ID (Biotage) and sequence analyses were carried out using the PyroQ-CpG analysis software (Biotage). All experiments included a human genomic DNA universally methylated for all genes (Intergen Company) as a positive control and water blanks as technical control.

***Cell Culture***

MM.1R, MM.1S, and KMS-11 MM cell lines were cultured in RPMI-1640 medium (Lonza) supplemented with 10% heat inactivated Fetal Bovine Serum (FBS) (Life Technologies), 1% Penicillin/Streptomycin (Biowhittaker) and 2% HEPES (Life Technologies). HEK-293T were maintained in DMEM (Lonza) with 10% of FBS, 1% of Penicillin/Streptomycin and 2% HEPES (Life Technologies). Only cells at low passage (below passage 15) were used for lentiviral production. All cell lines were maintained at 37 °C in a 5% CO_2_ humidified atmosphere. All cell lines were mycoplasma free and were authenticated by the Genomic Service of CIMA Lab Diagnostics of CIMA Universidad de Navarra.

***Knockdown systems***

Short hairpins RNAs (shRNA) against *SMILO* were designed using public available algorithms ([https://www.med.nagoya-u.ac.jp/neurogenetics/i_Score/i_score.html](about:blank) and [http://gesteland.genetics.utah.edu/siRNA_scales/](about:blank)). shRNAs were cloned into pLKO.1 vector (Addgene #8453). Lentiviruses were produced by transfecting HEK-293T cells grown in 150x25 mm plates at 70% of confluence, with 3 ug pMD2G (Addgene, #12259), 6 ug psPAX2 (Addgene, #12260) and 9 ug shRNA-expressing or scramble plasmid pLKO.1, using Lipofectamine® 2000 (Invitrogen) as instructed. After 72 hours, the supernatant was filtered (0.45 μm) and ultracentrifuged (Optima^tm^ LE-80K Ultracentrifuge, Beckman, California, USA) for 2.5 hours at 26,000 x g and 4 ºC. Pellets were resuspended in 1 mL of 1X PBS, incubated 1 hour on ice, and used to infect 10^6^ cells per 100 μL of virus. After 48 hours, MM cells were selected with puromycin (2 μg/ml) and were incubated 72 hours until we use them for the following assays.

***RT-qPCR***

For RNA extraction, 1 mL of TRIzol® Reagent (Life Technologies) was added to one million cells, and RNA was isolated following the manufacturer’s recommendations. One microgram of total RNA was used for reverse transcription using PrimeScript™ RT reagent Kit (Perfect Real Time) (Takara). qPCRs were performed using SYBR Green Master Mix with a QuantStudio 5 Real-Time PCR System (Thermo Fisher). The 2^-ΔΔCt^ method^16^ was used to calculate the relative expression level of transcripts normalized to *GUSß*. The sequence of primers used can be found in **Supplemental** **Table 6**.

***Proliferation and apoptosis assays***

To determine cell proliferation, 50,000 cells were cultured in triplicate for each condition in a 96-well plate, and, at the indicated time points, MTS assays were performed using CellTiter 96® Aqueous One Solution Cell Proliferation Assay kit (Promega) following the manufacturer's protocol. Absorbance was read at 490 nm with Sunrise™ (TECAN). Cell viability was calculated as the percentage of total absorbance of cells in each condition relative to control cells.

To determine the percentage of apoptotic cells, Annexin V-FITC assays were performed. Briefly, 100,000 cells were labelled with Annexin V and propidium iodide following manufacturer’s instructions (BD Biosciences Annexin V: PE Apoptosis Detection Kit I, Fisher Scientific), and analyzed by flow cytometry with a cytometer BD FACSCalibur^tm^ and FlowJo flow cytometry analysis software.

***Statistical analyses for qPCR, proliferation and apoptosis assays***

For both assays, statistical comparisons were performed using Shapiro-Wilk test for normality analyses, followed by Levenne test for homogeneity of variances. For parametric group comparisons one-way ANOVA was used, and Wilcoxon test was selected for non-parametric analyses. For multiple comparisons, Tukey correction was used for samples with homogenous variances and Tamhane’s T2 for variance heterogeneity.

***MARS-Seq of knockdown system samples***

Bulk RNA-seq was performed following MARS-Seq protocol adapted for bulk RNAseq^17,18^ with minor modifications. One microgram of RNA obtained from our *SMILO* knockdown systems was purified with DNase I RNase-free (Thermo Fisher). Briefly, 250 ng of poly-A RNA was reverse-transcribed using poly-dT oligos carrying a 7 bp-index. Pooled samples were subjected to linear amplification by IVT and resulting RNA was fragmented and dephosphorylated. Ligation of partial Illumina adaptor sequences^18^ was followed by a second RT reaction. Full Illumina adaptor sequences were added during final library amplification. RNA-seq libraries quantification was done with Qubit 3.0 Fluorometer (Life Technologies) and size profiles examination with Agilent’s 4200 TapeStation System. Libraries were sequenced in an Illumina NextSeq 500 at a sequence depth of 10 million reads per sample. MARS-seq data are available at GEO under accession number GSE134057. Raw reads were demultiplexed using bcl2fastq2, aligned to hg19 (bowtie2) and quantified (quant3p). Normalization and differential gene expression analyses were performed using the DEseq2 package (R). Gene ontology (GO) and Gene set Enrichment analyses (GSEA) were performed using the online tools from David Ontology and the Broad Institute, respectively. Results with false discovery rate (FDR) below 0.25 and a *P*-value below 0.05 were considered statistically significant.

***Interferon alpha treatment assays***

We treated three different MM cell lines (MM.1S, MM.1R and KMS-11) with 5 different concentrations of IFN alpha (IFNα): 10, 100, 500, 1000 and 5000 units/mL of IFNα (Sicor Biotech). After 24 h of treatment with IFNα, cells were collected to extract their RNA as previously described. We also collected cells at 0, 24, 48, 72 and 96 h to measure their proliferation by MTS; and at 48 and 96 h to measure the percentage of apoptosis by flow cytometry as previously described.

***Survival studies using the CoMMpass dataset***

For survival analyses, we used the survival data in the IA14 release of the Multiple Myeloma Research Foundation (MMRF) CoMMpass Study dataset. We used the progression-free survival (PFS) and overall survival (OS) data of 542 of MM patients. The difference between PFS and OS with all data was due to some NAs (Not Available) or incorrect values (such as negative survival). CoMMpass RNA-seq samples were processed with Salmon v0.9.1 using Gencode v27. We used the *maxStat* package (R) to select a threshold value for the expression of the lncRNAs. In order to avoid the bias introduced by this package, the threshold was selected as the median of 50,000 possible thresholds calculated with one third of the samples, randomly but balanced selected. Using the threshold, we discretized the lncRNAs as lowly or highly expressed, and then performed univariate COX regressions. We searched the synergy with biomarkers published in IA14, selected and filtered using *coxph* and *LogRank* single variable regression, selecting only those variables with significant p-value. These variables were included in a multivariate coxph regression in order to select the ones that remained significant. We have used high-risk genetic signatures, as well as several clinical data: gender, age (greater than 65), race, ISS stage and the applied treatment. We performed multivariate analyses with Backward Stepwise regression for PFS and OS.

**SUPPLEMENTAL REFERENCES:**

1. Dobin A, Davis CA, Schlesinger F, Drenkow J, Zaleski C, Jha S, *et al.* STAR: ultrafast universal RNA-seq aligner. *Bioinformatics.* 2013;29(1):15-21.

2. Harrow J, Frankish A, Gonzalez JM, Tapanari E, Diekhans M, Kokocinski F, *et al.* GENCODE: the reference human genome annotation for The ENCODE Project. *Genome Res.* 2012;22(9):1760-1774.

3. Trapnell C, Williams BA, Pertea G, Mortazavi A, Kwan G, van Baren MJ, *et al.* Transcript assembly and quantification by RNA-Seq reveals unannotated transcripts and isoform switching during cell differentiation. *Nat Biotechnol.* 2010;28(5):511-515.

4. Roberts A, Trapnell C, Donaghey J, Rinn JL, Pachter L. Improving RNA-Seq expression estimates by correcting for fragment bias. *Genome Biol.* 2011;12(3):R22.

5. Team RC. R: A Language and Environment for Statistical Computing. In:2013.

6. Tange O. Gnu parallel - the command-line power tool In*.* Vol 36: *The USENIX Magazine*; 2011.

7. Lin MF, Jungreis I, Kellis M. PhyloCSF: a comparative genomics method to distinguish protein coding and non-coding regions. *Bioinformatics.* 2011;27(13):i275-282.

8. Liao Y, Smyth GK, Shi W. featureCounts: an efficient general purpose program for assigning sequence reads to genomic features. *Bioinformatics.* 2014;30(7):923-930.

9. Love MI, Huber W, Anders S. Moderated estimation of fold change and dispersion for RNA-seq data with DESeq2. *Genome Biol.* 2014;15(12):550.

10. Li B, Ruotti V, Stewart RM, Thomson JA, Dewey CN. RNA-Seq gene expression estimation with read mapping uncertainty. *Bioinformatics.* 2010;26(4):493-500.

11. Zhu LJ, Gazin C, Lawson ND, Pagès H, Lin SM, Lapointe DS, *et al.* ChIPpeakAnno: a Bioconductor package to annotate ChIP-seq and ChIP-chip data. *BMC bioinformatics.* 2010;11:237.

12. Ritchie ME, Phipson B, Wu D, Hu Y, Law CW, Shi W, *et al.* limma powers differential expression analyses for RNA-sequencing and microarray studies. *Nucleic Acids Res.* 2015;43(7):e47.

13. Kohonen T. Self-Organized Formation of Topologically Correct Feature Maps. In*.* Vol 43: Biological Cybernetics. 1982:59-69.

14. Ordoñez R, Kulis M, Russiñol N, Chapaprieta V, Carrasco-Leon A, García-Torre B, *et al.* Chromatin activation as a unifying principle underlying pathogenic mechanisms in multiple myeloma. *Genome Reserach.* 2020;30(9):1217-1227.

15. Agirre X, Castellano G, Pascual M, Heath S, Kulis M, Segura V, *et al.* Whole-epigenome analysis in multiple myeloma reveals DNA hypermethylation of B cell-specific enhancers. *Genome Res.* 2015;25(4):478-87

16. Livak KJ, Schmittgen TD. Analysis of relative gene expression data using real-time quantitative PCR and the 2(-Delta Delta C(T)) Method. *Methods.* 2001;25(4):402-408.

17. Lavin Y, Kobayashi S, Leader A, Amir ED, Elefant N, Bigenwald C, *et al.* Innate Immune Landscape in Early Lung Adenocarcinoma by Paired Single-Cell Analyses. *Cell.* 2017;169(4):750-765 e717.

18. Jaitin DA, Kenigsberg E, Keren-Shaul H, Elefant N, Paul F, Zaretsky I, *et al.* Massively parallel single-cell RNA-seq for marker-free decomposition of tissues into cell types. *Science.* 2014;343(6172):776-779.

**SUPPLEMENTAL FIGURE LEGENDS**

**Supplemental Figure 1. lncRNAs expressed in MM. A)** Validation of the expression of 3 MM-identified lncRNAs in a new cohort of BMPC and MM samples. The data is shown as a CT values obtained by PCR. **B)** Violin plots representing the coefficient of variation (CV) of the expression of coding and each type of long non-coding transcripts in MM and BMPCs samples. Pairwise comparison employing Bonferroni test showed significant results (p < 2.2^e-16^) for each group of transcripts between MM and BMPCs specimens. **C)** Validation of 3 differentially expressed lncRNAs in a new cohort of BMPC and MM samples. MM=Multiple myeloma plasma cell; BMPC=Bone marrow plasma cell; G19lncRNAs=lncRNAs previously annotated in Gencode 19 database; BC-identified lncRNAs=lncRNAs identified in different B-cell populations on our previous work; and MM-identified lncRNAs=lncRNAs identified in MM patient samples.

**Supplemental Figure 2. Epigenetic regulation of specific MM lncRNAs. A)** Graphs depicting the percentage of the loci of lncRNAs (y axis) occupied by individual chromatin states. **B)** Heatmap showing the group of MM-identified lncRNAs that do not have *de novo* activation chromatin histone marks in MM. The color scale indicates the percentage of active chromatin sates in the promoter region of each lncRNA. LncRNAs from X and Y chromosomes were deleted in the analysis, remaining 860 of the previous 900 lncRNAs. **C)** Box plots representing the expression levels of lncRNAs from cluster 3 showing *de novo* epigenetic marks in each MM patient (orange) and those lncRNAs without such gain (purple). NB=Naïve; GC=Germinal center; MEM=Memory B-cell; TPC=Tonsil plasma cell; MM=Multiple myeloma plasma cell; Heterochrom.=Heterochromatin; chrom.=chromatin; *De novo*=lncRNAs with *de novo* chromatin active marks; Non-*de novo*=lncRNAs without *de novo* chromatin active marks.

**Supplemental Figure 3. lncRNAs expression in high-risk genetic subgroups of MM.** Expression levels (log2 (TPM+1)) of *ANKRD20A5P, SMILO, PDLIM1P4, ENSG00000249988, ENSG00000254343 and RHOT1P1* in MM subgroups of patients showing the significance of the presence (positive, red) or absence (negative, blue) of 4 high-risk genetic factors. P-values calculated with a two-sided t-test and adjusted for multiple hypotheses using FDR are presented for each comparison, with statistically significant values indicated in red. NEG=absence of alteration (blue); POS=presence of alteration (red).

**Supplemental Figure 4. *SMILO* is essential for the survival of MM cells. A)** Validation of the percentage of DNA methylation of two CpGs located in the promoter region of *SMILO* obtained from pyrosequencing assays in peripheral blood, B-cell populations and MM patient samples. CpG 1 (cg08458637) corresponds to methylation array data showed in **Figure 3A**; while CpG 2 corresponds to a CpG located next to CpG 1 and detected only by the pyrosequencing. The percentage of DNA methylation for each CpG is represented by a range of colors, from less DNA methylated (yellow) to 100% of DNA methylation (dark blue). **B)** Knockdown of *SMILO* by two different shRNAs in MM.1S cell lines. Levels of *SMILO* expression were determined by qPCR (left). Gene expression normalized to *GUSß* is presented relative to that observed in cells infected with a scramble shRNA. Proliferation curves (center) and the percentage of annexin-V positive cells (right) were detected at the indicated times after infection. Scramble represented in black, shRNA.A in orange and shRNA.B in blue. The average of three independent biological replicates +/- SD is shown. **C)** GSEA plots of the IFN pathway and RNA metabolism identified comparing KMS-11 cells with or without *SMILO* knockdown. **D)** Validation by qPCR of the overexpression of ISGs. **E-G)** The use of different concentrations of IFNα (10, 100, 500, 1000 and 5000u/ml) in MM cell lines (MM.1S, MM.1R and KMS-11) triggered the increase of apoptosis **(E)**, a decrease in proliferation **(F)** and the activation of the expression of different ISG **(G)** in a dose-dependent manner. **H)** Validation by qPCR of the overexpression of ERVs after inhibition of *SMILO* in MM.1S cells. *SMILO* knockdown samples were collected 5 days after infection. Samples treated with IFNα were collected after 24 h for qPCR analysis; at 0, 24, 48, 72 and 96 h to measure proliferation; and at 48 and 96 h to measure apoptosis. All gene expressions were normalized to *GUSß*, and were presented relative to that observed in cells infected with a scramble shRNA or to control cells. The average of three independent biological replicates +/- SD is shown. PB=Peripheral blood; BMPC=Bone marrow plasma cell; MM=Multiple myeloma plasma cell; NB=Naïve; CB=Centroblast; CC=Centrocyte; MEM=Memory B-cell; TPC=Tonsil plasma cell; FDRq=False discovery rate; NES=Normalized enrichment score; u=Concentration of IFNα in units/ml.

**Supplemental Figure 5. Analysis of the prognostic value of lncRNAs in MM. A)** CoMMpass expression data of the Gencode annotated lncRNAs obtained in the epigenomic study showed in **Figure 2F**. Expression is represented in log2(TPM+1). **B)** Histograms representing the selected thresholds over gene expression (up); and representing the empirical population of possible thresholds to dichotomize the lncRNAs expression (down). Threshold represented in red and quartiles in blue. Expression is represented in log2(TPM+1).

**Supplemental Figure 6.** **Progression-free survival and overall survival probability of lncRNAs in MM.** **A-F)** Kaplan-Meier curves represent a bi-level state of expression (high and low) of the lncRNAs showing the no significant p-value in terms of progression-free survival **(A-C)** and overall survival **(D-F)**. LOW=low expression of the lncRNA; HIGH=high expression of the lncRNA.

**SUPPLEMENTAL TABLES**

**Supplemental Table 1. MM patients data used for the ssRNA-seq.** Del=Deletion; Amp=Amplification; MM=Multiple myeloma; POS=Positive; NEG=Negative; NA=Not available.

**Supplemental Table 2. Expression of MM-identified lncRNAs in MM patient samples**. TPM values from ssRNA-Seq of MM patient samples are shown. Chr=Chromosome.

**Supplemental Table 3. Percentage of lncRNAs deregulated in MM patients.** NA=Not Available; Up=Upregulated; Down=Downregulated.

**Supplemental Table 4. Expression data of lncRNAs with exclusive expression in MM patients (Cluster 3).** Expression values in log2FPKM. NA=Not available.

**Supplemental Table 5. lncRNAs with *de novo* active chromatin marks in MM.** NA=Not Available.

**Supplemental Table 6. Primer sequences.** Fw=Forward primers; Rv=Reverse primers; *Bio=Biotinylated.
