# Supplementary material for: Characterization of complete lncRNAs transcriptome reveals the functional and clinical impact of lncRNAs in multiple myeloma
Source: Leukemia. 2021 Feb 17;35(5):1438–50. doi: 10.1038/s41375-021-01147-y (PMC8102198; doi:10.1038/s41375-021-01147-y)
Supplement: Supplementary file 2 — Supplemental Figures [file 41375_2021_1147_MOESM2_ESM.pptx]

## Slide 1
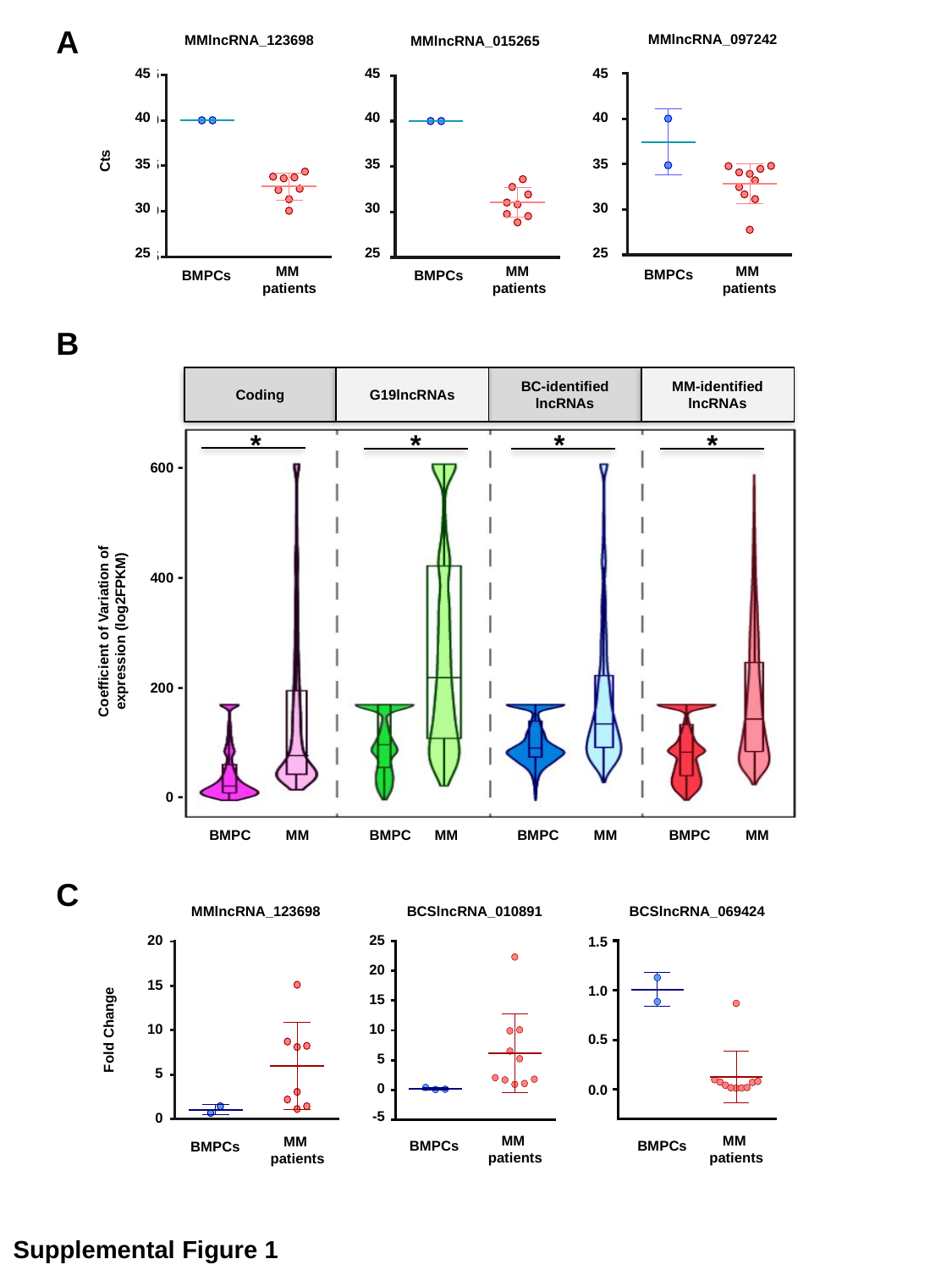

A
MMlncRNA_097242
45
40
35
30
25
MM
patients
BMPCs
MMlncRNA_123698
45
40
35
30
25
MM
patients
BMPCs
Cts
MMlncRNA_015265
45
40
35
30
25
MM
patients
BMPCs
B
Coding
G19lncRNAs
BC-identified lncRNAs
MM-identified lncRNAs
600 -
400 -
200 -
0 -
Coefficient of Variation of expression (log2FPKM)
BMPC
MM
BMPC
MM
BMPC
MM
BMPC
MM
*
*
*
*
C
MMlncRNA_123698
20
15
10
5
0
Fold Change
MM
patients
BMPCs
BCSlncRNA_010891
25
20
15
10
5
0
-5
MM
patients
BMPCs
BCSlncRNA_069424
1.5
1.0
0.5
0.0
MM
patients
BMPCs
Supplemental Figure 1

## Slide 2
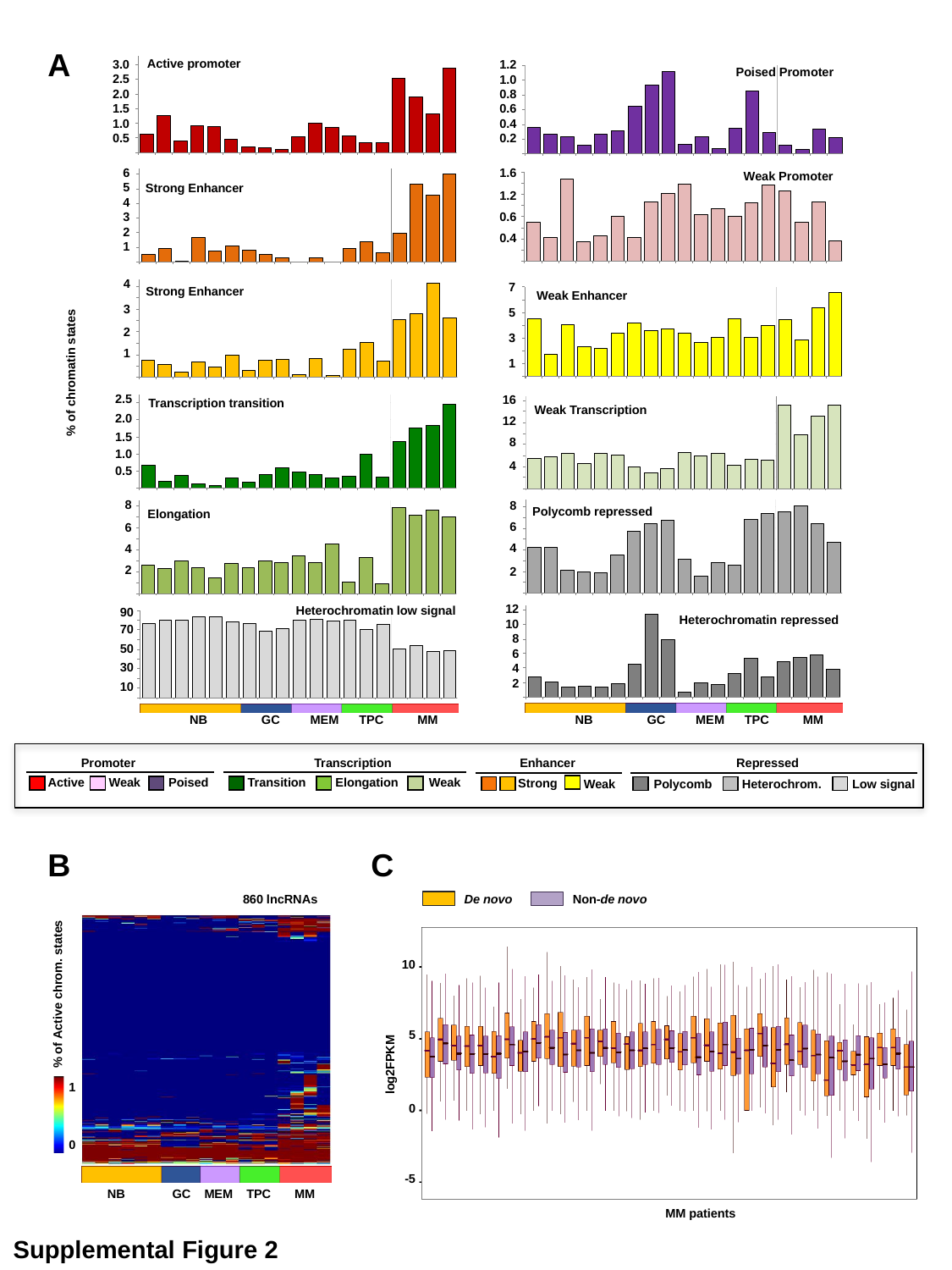

A
Active promoter
3.0
2.5
2.0
1.5
1.0
0.5
1.2
1.0
0.8
0.6
0.4
0.2
Poised Promoter
1.6
1.2
0.6
0.4
Weak Promoter
6
5
4
3
2
1
Strong Enhancer
4
3
2
1
Strong Enhancer
7
5
3
1
Weak Enhancer
% of chromatin states
2.5
2.0
1.5
1.0
0.5
Transcription transition
16
12
8
4
Weak Transcription
8
6
4
2
Elongation
8
6
4
2
Polycomb repressed
12
10
8
6
4
2
Heterochromatin repressed
Heterochromatin low signal
90
70
50
30
10
NB GC MEM TPC MM
NB GC MEM TPC MM
Promoter
Active
Weak
Poised
Transcription
Transition
Elongation
Weak
Enhancer
Strong
Weak
Repressed
Polycomb
Heterochrom.
Low signal
C
B
860 lncRNAs
NB GC MEM TPC MM
% of Active chrom. states
1
0
Non-de novo
De novo
10
5
0
-5
log2FPKM
MM patients
Supplemental Figure 2

## Slide 3
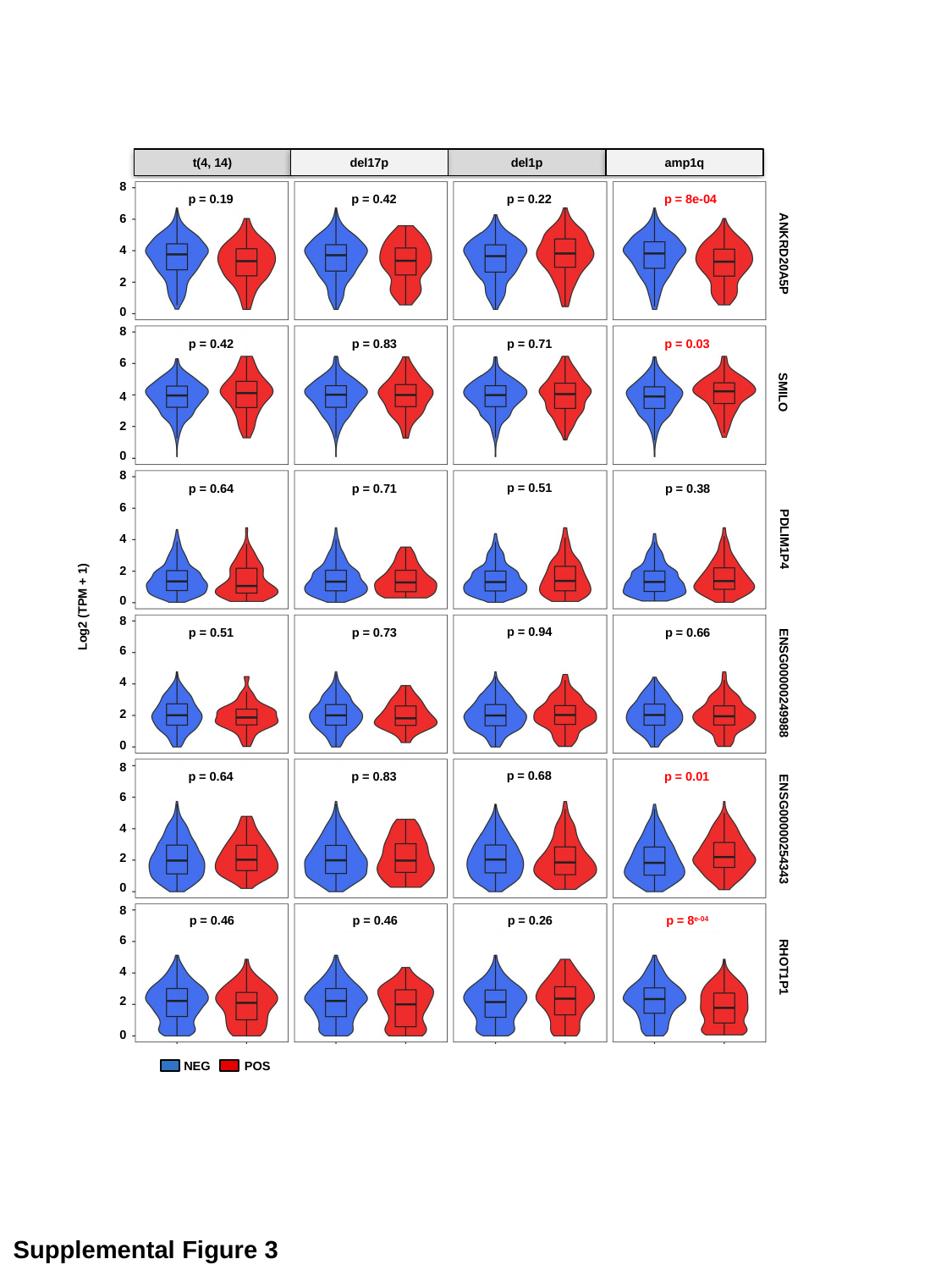

t(4, 14)
del17p
del1p
amp1q
8
6
4
2
0
p = 0.22
p = 0.19
p = 0.42
p = 8e-04
ANKRD20A5P
8
6
4
2
0
p = 0.71
p = 0.42
p = 0.83
p = 0.03
SMILO
8
6
4
2
0
p = 0.51
p = 0.38
p = 0.64
p = 0.71
PDLIM1P4
Log2 (TPM + 1)
8
6
4
2
0
p = 0.94
p = 0.66
p = 0.51
p = 0.73
ENSG00000249988
8
6
4
2
0
p = 0.68
p = 0.64
p = 0.83
p = 0.01
ENSG00000254343
8
6
4
2
0
p = 0.26
p = 0.46
p = 0.46
p = 8e-04
RHOT1P1
NEG
POS
Supplemental Figure 3

## Slide 4
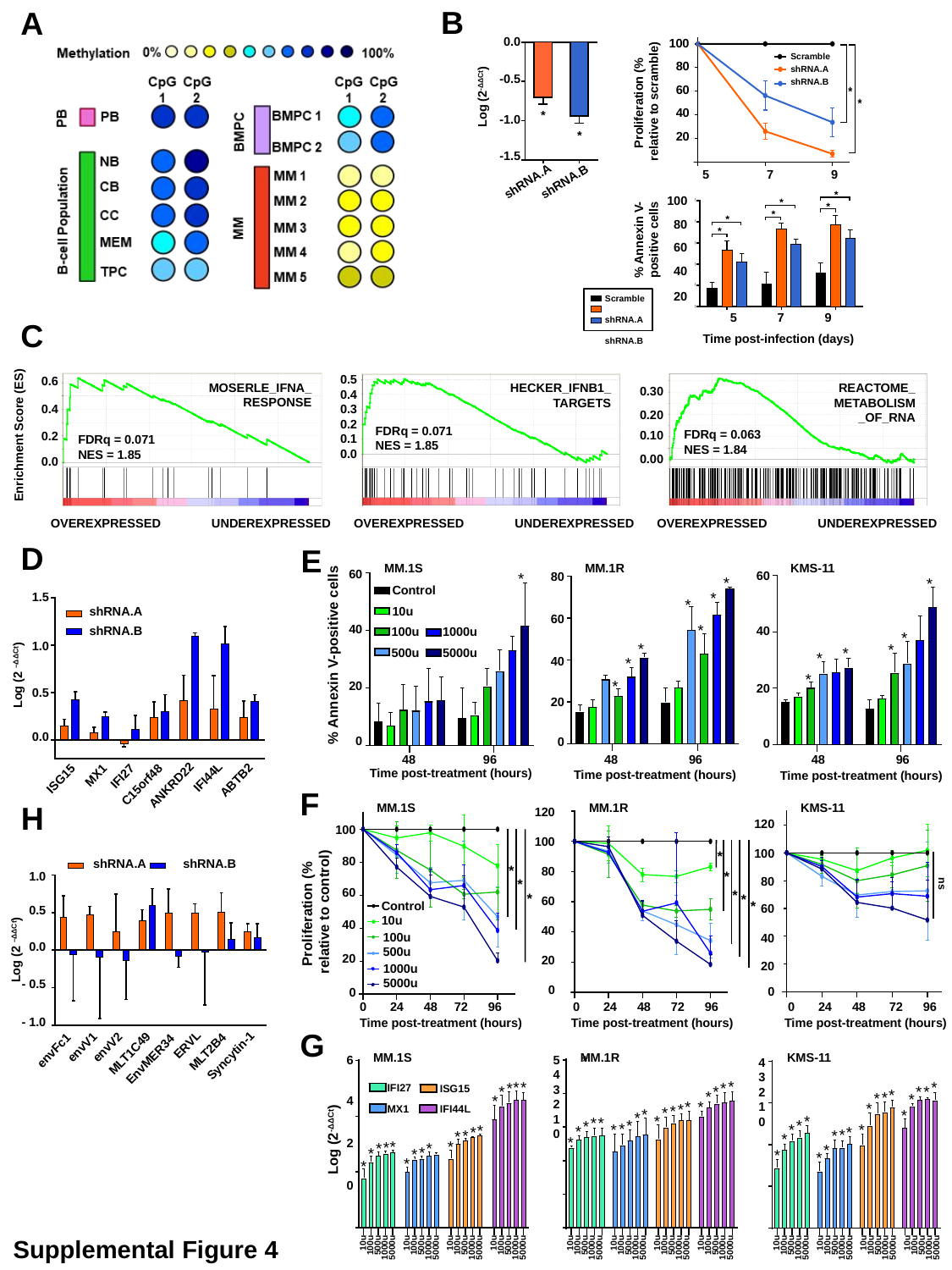

B
A
100
80
60
40
20
Scramble
shRNA.A
shRNA.B
Proliferation (%
relative to scramble)
5 7 9
0.0
-0.5
-1.0
-1.5
Log (2-ΔΔCt)
shRNA.A
shRNA.B
*
*
100
80
60
40
20
% Annexin V-positive cells
5 7 9
Time post-infection (days)
Scramble
shRNA.A
shRNA.B
C
0.6
0.4
0.2
0.0
MOSERLE_IFNA_RESPONSE
FDRq = 0.071
NES = 1.85
Enrichment Score (ES)
OVEREXPRESSED UNDEREXPRESSED
0.5
0.4
0.3
0.2
0.1
0.0
HECKER_IFNB1_TARGETS
FDRq = 0.071
NES = 1.85
OVEREXPRESSED UNDEREXPRESSED
REACTOME_METABOLISM_OF_RNA
0.30
0.20
0.10
0.00
FDRq = 0.063
NES = 1.84
OVEREXPRESSED UNDEREXPRESSED
D
E
MM.1S
60
40
20
0
*
Control
10u
100u
500u
1000u
5000u
% Annexin V-positive cells
48 96
Time post-treatment (hours)
MM.1R
80
60
40
20
0
*
*
*
*
*
*
*
48 96
Time post-treatment (hours)
KMS-11
60
40
20
0
*
*
*
*
*
*
48 96
Time post-treatment (hours)
F
MM.1S
MM.1R
120
100
80
60
40
20
0
*
*
*
*
*
0 24 48 72 96
Time post-treatment (hours)
KMS-11
120
100
80
60
40
20
0
ns
0 24 48 72 96
Time post-treatment (hours)
100
80
60
40
20
0
*
*
*
Proliferation (%
relative to control)
Control
10u
100u
500u
1000u
5000u
0 24 48 72 96
Time post-treatment (hours)
G
MM.1S
6
4
2
0
*
*
*
IFI27
MX1
ISG15
IFI44L
*
*
*
*
*
*
Log (2-ΔΔCt)
*
*
*
*
*
*
*
*
*
*
10u
100u
500u
1000u
5000u
10u
100u
500u
1000u
5000u
10u
100u
500u
1000u
5000u
10u
100u
500u
1000u
5000u
MM.1R
*
5
4
3
2
1
0
*
*
*
*
*
*
*
*
*
*
*
*
*
*
*
*
*
*
*
*
10u
100u
500u
1000u
5000u
10u
100u
500u
1000u
5000u
10u
100u
500u
1000u
5000u
10u
100u
500u
1000u
5000u
KMS-11
4
3
2
1
0
*
*
*
*
*
*
*
*
*
*
*
*
*
*
*
*
*
*
*
*
10u
100u
500u
1000u
5000u
10u
100u
500u
1000u
5000u
10u
100u
500u
1000u
5000u
10u
100u
500u
1000u
5000u
1.5
1.0
0.5
0.0
shRNA.A
shRNA.B
Log (2 –ΔΔCt)
MX1
IFI44L
ISG15
IFI27
ABTB2
C15orf48
ANKRD22
H
shRNA.A shRNA.B
1.0
0.5
0.0
- 0.5
- 1.0
Log (2 –ΔΔCt)
ERVL
envV1
envV2
envFc1
MLT2B4
MLT1C49
Syncytin-1
EnvMER34
Supplemental Figure 4

## Slide 5
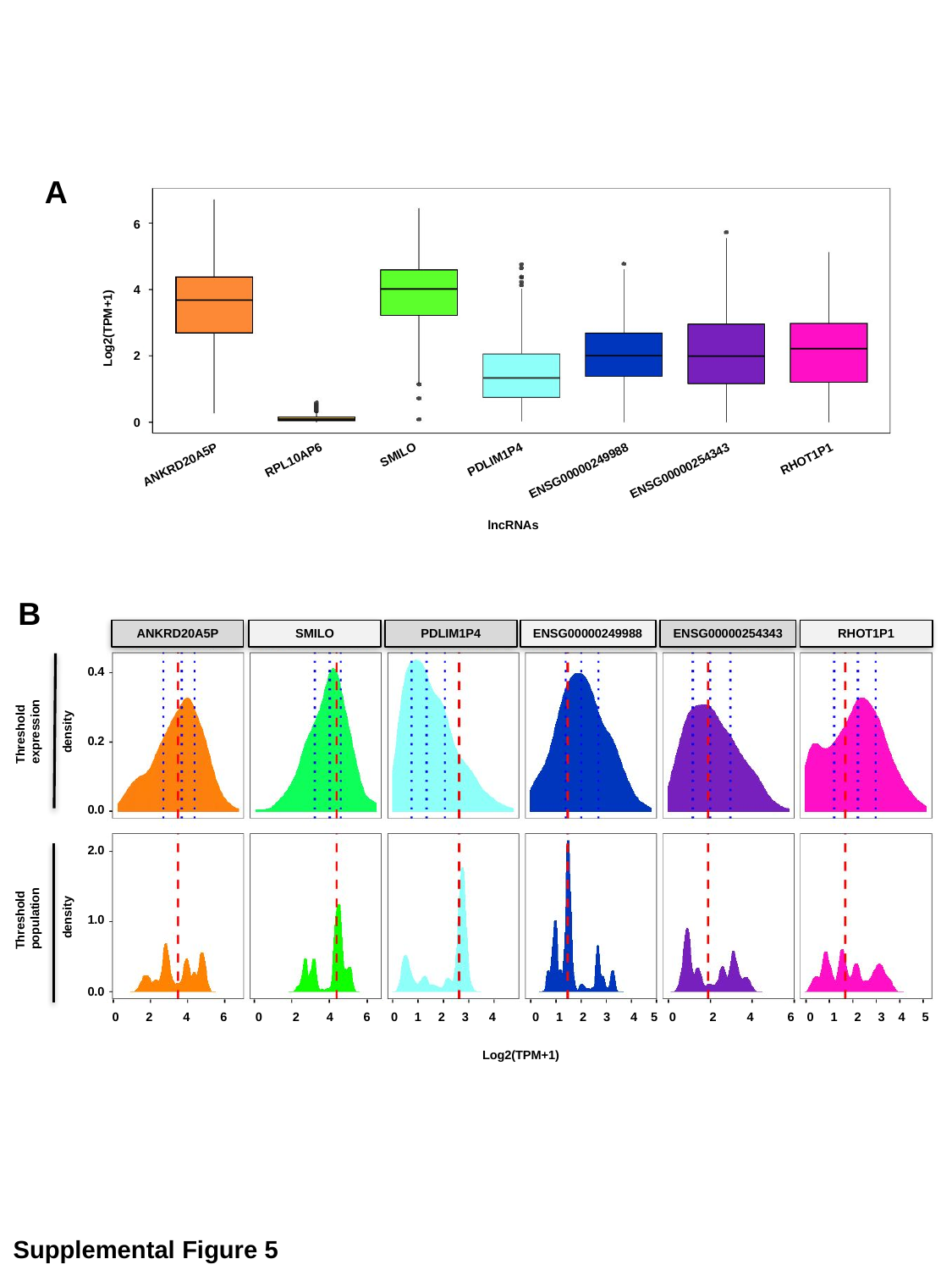

A
6
4
2
0
Log2(TPM+1)
SMILO
RHOT1P1
PDLIM1P4
RPL10AP6
ANKRD20A5P
ENSG00000249988
ENSG00000254343
lncRNAs
B
ANKRD20A5P
SMILO
PDLIM1P4
ENSG00000249988
ENSG00000254343
RHOT1P1
0.4
0.2
0.0
Threshold
expression
density
2.0
1.0
0.0
Threshold
population
density
0 2 4 6
0 2 4 6
0 1 2 3 4
0 1 2 3 4 5
0 2 4 6
0 1 2 3 4 5
Log2(TPM+1)
Supplemental Figure 5

## Slide 6
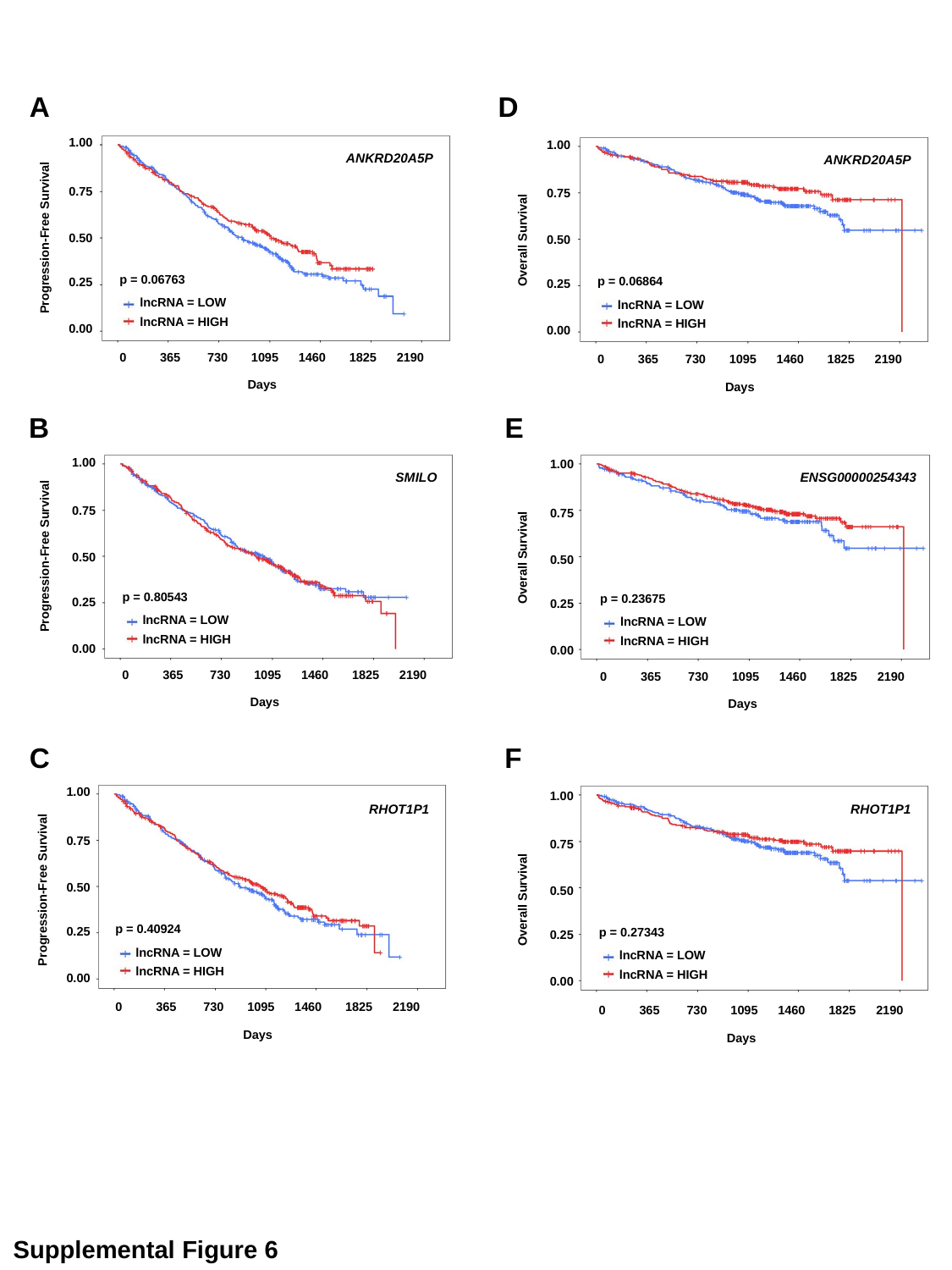

A
D
1.00
0.75
0.50
0.25
0.00
ANKRD20A5P
Progression-Free Survival
p = 0.06763
lncRNA = LOW
lncRNA = HIGH
0 365 730 1095 1460 1825 2190
Days
1.00
0.75
0.50
0.25
0.00
ANKRD20A5P
Overall Survival
p = 0.06864
lncRNA = LOW
lncRNA = HIGH
0 365 730 1095 1460 1825 2190
Days
B
E
1.00
0.75
0.50
0.25
0.00
SMILO
Progression-Free Survival
p = 0.80543
lncRNA = LOW
lncRNA = HIGH
0 365 730 1095 1460 1825 2190
Days
1.00
0.75
0.50
0.25
0.00
ENSG00000254343
Overall Survival
p = 0.23675
lncRNA = LOW
lncRNA = HIGH
0 365 730 1095 1460 1825 2190
Days
C
F
1.00
0.75
0.50
0.25
0.00
RHOT1P1
Progression-Free Survival
p = 0.40924
lncRNA = LOW
lncRNA = HIGH
0 365 730 1095 1460 1825 2190
Days
1.00
0.75
0.50
0.25
0.00
RHOT1P1
Overall Survival
p = 0.27343
lncRNA = LOW
lncRNA = HIGH
0 365 730 1095 1460 1825 2190
Days
Supplemental Figure 6
